# Regulatory (FoxP3^+^) T cells and TGF-β predict the response to anti-PD-1 immunotherapy in patients with non-small cell lung cancer

**DOI:** 10.1038/s41598-020-76130-1

**Published:** 2020-11-04

**Authors:** Jiae Koh, Joon Young Hur, Kyoung Young Lee, Mi Soon Kim, Jae Yeong Heo, Bo Mi Ku, Jong-Mu Sun, Se-Hoon Lee, Jin Seok Ahn, Keunchil Park, Myung-Ju Ahn

**Affiliations:** 1 Department of Health Sciences and Technology, SAIHST, Sungkyunkwan University, 81 Irwon-ro, Gangnam-gu, Seoul, Republic of Korea; 2 Division of Hematology-Oncology, Department of Medicine, Samsung Medical Center, Sungkyunkwan University School of Medicine, Seoul, Republic of Korea; 3 Research Institute for Future Medicine, Samsung Medical Center, Sungkyunkwan University School of Medicine, Seoul, Republic of Korea; 4 Division of Hematology and Oncology, Department of Internal Medicine, Hanyang University Guri Hospital, Guri, Republic of Korea

**Keywords:** Cancer, Immunology, Biomarkers, Oncology

## Abstract

Antitumor immune responses induced by immune checkpoint inhibitors anti-PD-1 or anti-PD-L1 have been used as therapeutic strategies in advanced non-small cell lung cancer (NSCLC) patients over the last decade. Favorable antitumor activity to immune checkpoint inhibitors is correlated with high PD-L1 expression, increased tumor-infiltrating lymphocytes, and decreased suppressive immune cells including Treg cells, myeloid-derived suppressor cells, or tumor-associated macrophages in various cancer types. In this study, we investigated the potential correlation between clinical outcomes and peripheral blood immune cell profiles, specifically focused on FoxP3^+^ Treg cells, collected at baseline and one week after anti-PD-1 therapy in two independent cohorts of patients with NSCLC: a discovery cohort of 83 patients and a validation cohort of 49 patients. High frequencies of circulating Treg cells one week after anti-PD-1 therapy were correlated with a high response rate, longer progression-free survival, and overall survival. Furthermore, high levels of TGF-β and Treg cells were associated with favorable clinical outcomes. Our results suggest that higher levels of FoxP3^+^ Treg cells and TGF-β can predict a favorable response to anti-PD-1 immunotherapy in patients with advanced NSCLC.

## Introduction

During the past decade, immunotherapy targeting T cell inhibitory receptors has rapidly emerged as a frontline therapeutic option in advanced non-small cell lung cancer (NSCLC). Nivolumab or pembrolizumab targeting programmed cell death-1 (PD-1) and atezolizumab or durvalumab targeting programmed cell death-ligand 1 (PD-L1) have been approved^1,2^. However, a durable response to immune checkpoint inhibitors as a single agent is observed in less than 20% of NSCLC patients, and the immune mechanisms involved in the response to this therapy and in the development of resistance, which significantly limits broader clinical application, remain poorly understood^3,4^.

Tumor microenvironments can be a hurdle for therapeutic effects limiting T cell infiltration and activation. Immunosuppressive mechanisms in tumors are usually associated with the up-regulation of molecules that decrease T cell function. Suppressive immune cells including polymorphonuclear (PMN)-myeloid-derived suppressor cells (MDSCs), monocytic (M)- MDSCs, tumor-associated macrophages (TAMs), and regulatory T cells (Tregs), and cytokines or small molecules released by these cells such as TGF-β, interleukin (IL)-10, IL-6, or vascular endothelial growth factor (VEGF), are also involved in the suppressive microenvironment in tumor sites^5–8^.

Among suppressive immune cells, Treg cells are a subset of T cells expressing IL-2 receptor CD25 and forkhead/flanking helix nuclear transcription factor (FoxP3). Treg cells regulate immune responses in the body by maintaining immune homeostasis and preventing the development of autoimmune diseases^9^. Treg cells play a role in the immunosuppression of tumor tissue by promoting the differentiation, proliferation, and secretion of immunosuppressive factors^10,11^. Treg cells constitute 5–10% of total peripheral CD4^+^ T cells, and a high frequency of Treg cells at tumor sites has negative impacts on the outcomes of cancer patients^12^.

TGF-β is a cytokine released by Treg cells, and the immune-suppressive functions of Tregs and TGF-β are acknowledged widely and have been researched extensively. Nonetheless, recent studies have revealed the positive roles of Treg cells and TGF-β in shaping the immune system and inflammatory responses in advanced colon cancer, HPV-positive oral and oropharyngeal squamous cell carcinomas, and estrogen receptor-negative breast cancer^13–15^. Treg cells and TGF-β may also positively regulate immune responses depending on tumor types or in different tumor microenvironments. Thus, while Treg cells and TGF-β are dominantly viewed as critical mediators for immune suppression, they exert both negative and positive effects on the immune system^16,17^.

To understand the dynamic changes and functional interplays of immune cells in the tumor microenvironment, the analysis of circulating immune cells, as a non-invasive alternative source, will provide an important therapeutic insight after immunotherapy. Moreover, it is important to identify patients who may benefit from immune checkpoint inhibitors and to discriminate responders from non-responders before the initiation of treatment or at an early point in the therapy.

In this study, we analyzed the frequency of circulating Treg cells one week after anti-PD-1 immunotherapy and their correlation alone or together with other suppressive immune cells and clinical outcomes including progression-free survival (PFS) and overall survival (OS) in advanced NSCLC patients. We also analyzed TGF-β possibly released by Treg cells and its correlation with clinical outcomes to evaluate its role as a potential biomarker to predict response to anti-PD-1 therapy.

## Results

### Clinical outcomes of anti-PD-1 immunotherapy associated with Treg cell frequencies before and after therapy

**Figure 1:**
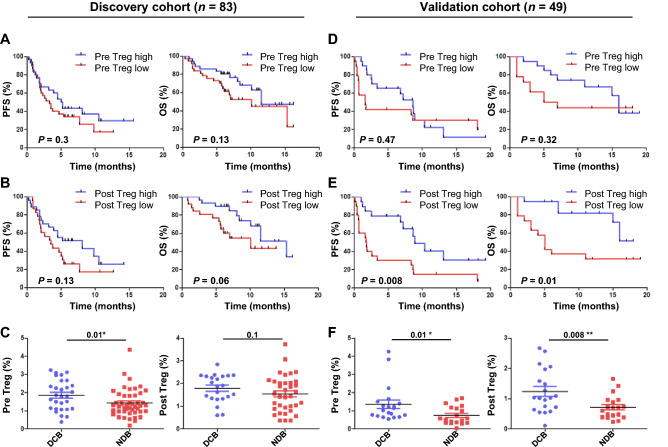
Progression-free survival (PFS) and overall survival (OS) of patients with advanced NSCLC in association with Treg cell frequencies. (**A**) PFS and OS in relation to high or low frequencies of Treg cells before and (**B**) after one week of anti-PD-1 therapy. (**C**) Treg cell frequencies of durable clinical benefiters (DCB) or non-durable benefiters (NDB) pre- and post-therapy in the discovery cohort (*n* = 83). PFS and OS in association with Treg cells frequencies (**D**) before and (**E**) after one week of anti-PD-1 therapy. (**F**) Treg cell frequencies of patients with DCB or NDB in the validation cohort (*n* = 49). Treg cells before the therapy, the median cutoff values of pre-Treg cells were 1.41% (range 0.18–4.36) in the discovery cohort and 0.84% (range 0.04–4.26) in the validation cohort. After the therapy, the median cutoff values of post-Treg were 1.6% (range 0.37–3.73) in the discovery cohort and 0.75% (range 0.11–2.67) in the validation cohort. Center value mean ± SEM of pre-Treg cells are 1.86 (DCB, *n* = 29), and 1.43 (NDB, *n* = 46), and post-Treg cells are 1.79 (DCB, *n* = 22) and 1.54 (NDB, *n* = 36) in (**C**). Center value mean ± SEM of pre-Treg cells are 1.365 (DCB, *n* = 20) and 0.755 (NDB, *n* = 19), and post-Treg cells are 1.239 (DCB, *n* = 20) and 0.5211 (NDB, *n* = 19) in (**F**). Kaplan–Meier survival curves of patients were plotted with a median cutoff. Statistical significance was determined by log-rank (Mantel-Cox) regression analysis, with the level of significance at *P* $\le$ 0.05.

To analyze effector Treg cells and their correlation with clinical outcomes before and after anti-PD-1 immunotherapy, the levels of circulating Treg cells were determined by flow cytometer in peripheral blood mononuclear cells (PBMCs) and patients were categorized into low and high Treg cell groups with a median cutoff value. Circulating Treg cells were defined as CD4^+^CD25^+^CD45RA^-^FoxP3^+^ (effector Treg cells) and gating strategies are shown in Supplementary Fig. S1. Patient characteristics are shown in Table 1. In the discovery cohort (*n* = 83), with a median follow-up duration of 11.5 months (range 0.4–31.5 months), patients with a high frequency of Treg cells showed a trend toward longer PFS and OS although the differences were not statistically significant both before (*P*_PFS_ = 0.3; 3.3 months vs. 5.9 months, *P*_OS_ = 0.13; 8.7 months vs. 14 months) (Fig. 1A) and after therapy (*P*_PFS_ = 0.13; 3.9 months vs 5.9 months, *P*_OS_ = 0.06; 13.8 months vs 11.5 months) (Fig. 1B). In addition, patients with a high frequency of Treg cells before therapy had more durable clinical benefits than the low group (*P*_pre_ = 0.01) compared with after therapy (*P*_post_ = 0.1) (Fig. 1C). Similar findings were observed in the validation cohort (*n* = 49). With a median follow-up of 6.6 months (range 0.2–19.3 months) there was no statistical significance between high and low Treg cell groups of the patients before therapy in PFS and OS (*P*_PFS_ = 0.47; 1.6 months vs. 6.8 months, *P*_OS_ = 0.32; 4.8 months vs. 9.9 months) (Fig. 1D). In contrast, one week after anti-PD-1 therapy, the group with a high frequency of Treg cells had a significantly longer PFS (*P* = 0.008; 1.7 months vs. 7.9 months) and OS (*P* = 0.01; 4.6 months vs. 12.3 months) compared to those with a low frequency of Treg cells (Fig. 1E). Also, both before and after therapy, the patients with a high frequency of Treg cells had greater durable clinical benefits than those with a low frequency of Treg cells (*P*_pre_ = 0.01, *P*_post_ = 0.008) (Fig. 1F). In addition to Treg cells with CD4^+^CD25^+^CD45RA^-^FoxP3^+^, we further analyzed Treg cells with CD4^+^CD25^+^FoxP3^+^ markers either from PBMCs (Supplementary Fig. S3A,B) or from CD4^+^ T cells (Supplementary Fig. S3C,D). Similar results were obtained from CD4^+^CD25^+^FoxP3^+^ Treg and effector Treg cells (CD4^+^CD25^+^CD45RA^-^FoxP3^+^) analyzed from PBMC, but the Treg cell percentage within CD4^+^ T cells was not significant in discovery or validation cohorts. Also, there was no significant difference in PFS or OS in the groups of patients with increased or decreased Treg cells in both discovery and validation cohorts (Supplementary Fig. S4). Collectively, patients with high frequencies of Treg cells had longer PFS and OS. In particular, patients with a high frequency of Treg cells one week after anti-PD-1 therapy had the most distinct differences.

**Table 1:** Characteristics of the patients with NSCLC.

Characteristics	Discovery cohort (*n* = 83)	Validation cohort (*n* = 49)
**Age—yr**		
Median	62	62
Range	39–88	34–82
**Gender no. (%)**		
Male	68 (81.9)	37 (75.5)
Female	15 (18.1)	12 (24.5)
**ECOG performance-status score—no. (%)**		
0	0 (0)	2 (4)
1	74 (89.2)	39 (79.6)
≥ 2	9 (10.8)	8 (16.4)
**Tumor histologic type—no. (%)**		
Adenocarcinoma	48 (57.8)	25 (51)
Squamous cell carcinoma	26 (31.3)	13 (26.5)
Others	9 (10.9)	11 (22.5)
**PD-L1 expression level – no. (%)**		
< 1%	15 (18)	8 (16.3)
≥ 1%	57 (68.7)	27 (55.1)
Unknown	11 (13.3)	14 (28.6)

*ECOG* Eastern Cooperative Oncology Group.

### Correlation of Treg cell frequency with MDSCs

**Figure 2:**
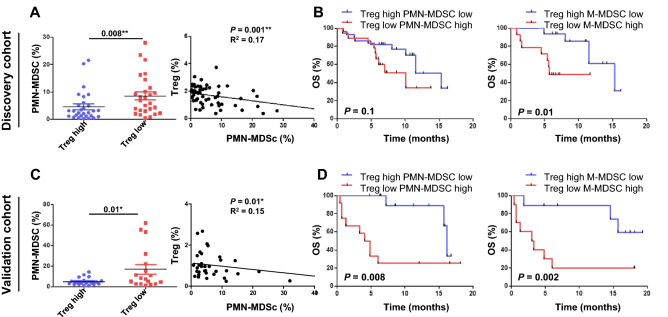
Correlation of Treg cells and myeloid-derived suppressor cells (MDSCs). (**A**) polymorphonuclear (PMN)-MDSC frequencies in association with high or low levels of Treg cells and coefficient correlation of Treg cells and PMN-MDSC frequencies in the discovery cohort. (**B**) Overall survival (OS) when levels of Treg cells are high and PMN-MDSCs are low and when levels of Treg cells are high and monocytic (M)-MDSCs are low compared with low levels of Treg cells and high levels of MDSCs in the discovery cohort. (**C**) PMN-MDSC frequencies in association with high or low levels of Treg cells and the coefficient correlation of Treg cells and PMN-MDSC frequencies in the validation cohort. (**D**) OS with high levels of Treg cells and low levels of PMN-MDSCs or M-MDSCs compared with low levels of Treg cells and high levels of MDSCs in the validation cohort. The median cutoff values of Treg cells are 1.6% (range 0.37–3.73) in discovery and 0.75% (range 0.11–2.67) in the validation cohort. The median cutoff values for PMN-MDSCs are 3.9% (range 0.22–70.55) in the discovery cohort and 4.4% (range 0.3–62) in the validation cohort. The median cutoff values for M-MDSCs are 11.1% (range 3–29.34) in the discovery cohort and 6.7% (range 1.5–30.8) in the validation cohort. High Treg cells and low PMN-MDSCs: discovery *n* = 19, validation *n* = 12. Low Treg cells and high PMN-MDSCs: discovery *n* = 18, validation *n* = 12. High Treg cells and low M-MDSCs: discovery *n* = 16, validation *n* = 9. Low Treg cells and high M-MDSCs: discovery *n* = 14, validation *n* = 10. The center value is the mean ± SEM. Kaplan–Meier survival curves were plotted with a median cutoff. Statistical significance was determined by log-rank (Mantel-Cox) regression analysis, with the level of significance at *P* $\le$ 0.05.

In a previous study, we reported that a low level of preexisting peripheral PMN-MDSCs, M-MDSCs, and CD39^+^CD8^+^ T cells correlate with favorable clinical outcomes in patients with advanced NSCLC^18^. Of note, in the current study, patients with high frequencies of Treg cells had relatively low PMN-MDSCs in their peripheral blood (*P* = 0.008) with a correlation coefficient value of *P* = 0.001 (r^2^ = 0.17) (Fig. 2A). However, high frequencies of Treg cells and low M-MDSCs showed no statistical significance (Supplementary Fig. S5A). Furthermore, in the discovery cohort, patients with a high frequency of Treg cells and a low frequency of PMN-MDSCs (*P*_OS_ = 0.1) or M-MDSCs (*P*_OS_ = 0.01) had better OS compared with those with a low frequency of Treg cells and high frequency of MDSCs (Fig. 2B). Similarly, in the validation cohort, patients with a high frequency of Treg cells had a low frequency of PMN-MDSCs compared with low Treg cells (*P* = 0.01) with a correlation coefficient value of *P* = 0.01 (r^2^ = 0.15) (Fig. 2C). However, high frequencies of Treg cells and M-MDSCs showed no statistical significance (Supplementary Fig. S5B). Patients with a high frequency of Treg cells and a low frequency of PMN-MDSCs (*P*_OS_ = 0.008) or M-MDSCs (*P*_OS_ = 0.002) had prolonged survival (Fig. 2D). Taken together, the group with a high frequency of Treg cells was associated with relatively low PMN-MDSC frequencies. Patients with the combination of high Treg cells and low PMN-MDSCs or M-MDSC had longer OS compared to those with low Treg cells and high PMN-MDSCs or M-MDSCs.

### TGF-β mRNA expression correlated with Treg cells and clinical outcomes

**Figure 3:**
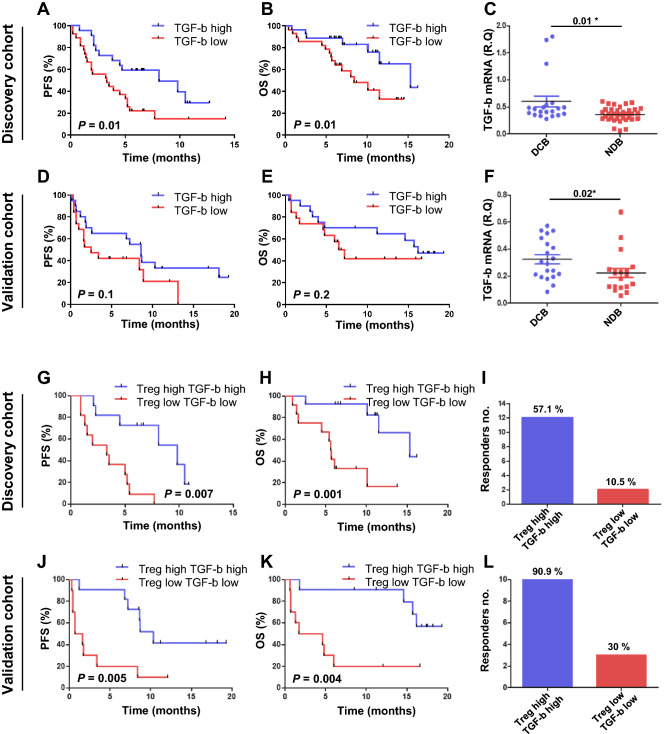
Cytokine mRNA levels and correlation with Treg cell frequencies. (**A**) Progression-free survival (PFS) and (**B**) overall survival (OS) associated with TGF-β mRNA level in the discovery cohort. (**C**) TGF-β mRNA levels of durable clinical benefiters (DCB) or non-durable clinical benefiters (NDB). (**D**) PFS and (**E**) OS associated with TGF-β mRNA level in the validation cohort. (**F**) TGF-β mRNA levels of DCB or NDB. (**G**) PFS and (**H**) OS of patients with both high levels of Treg cells and TGF-β compared to those with low levels of Treg cells and TGF-β. (**I**) The number of responders with high levels of Treg cells and TGF-β compared to those with low levels of Treg cells and TGF-β in the discovery cohort. (**J**) PFS and (**K**) OS of patients with both high levels of Treg cells and TGF-β compared to patients with low levels of Treg cells and TGF-β. (**L**) The number of responders associated with high levels of Treg cells and TGF-β compared to those with low levels of Treg cells and TGF-β in the validation cohort. The center value is the mean ± SEM. Patient survival curves were plotted with Kaplan–Meier by median cutoff. Statistical significance was determined by log-rank (Mantel-Cox) regression analysis, with the level of significance at *P* $\le$ 0.05. R.Q., relative quantification.

We next analyzed the mRNA expression of various cytokines including TGF-β, IL-10, and IL-6 one week after anti-PD-1 immunotherapy. Unlike other cytokines, patients with a high expression of TGF-β had a longer PFS (*P* = 0.01; 6.3 months vs. 15.2 months), OS (*P* = 0.01; 2.5 months vs. 8.6 months), and longer clinical benefits (*P* = 0.01) compared with patients with a low expression of TGF-β in the discovery cohort (Fig. 3A–C). In the validation cohort, a high level of TGF-β mRNA correlated with clinical benefits (*P* = 0.02) (Fig. 3D–F). Unlike TGF-β, low IL-10 expression one week after therapy correlated with longer PFS (*P* = 0.02) and OS (*P* = 0.002) when compared to those with high expression in the validation cohort (Supplementary Fig. S6A,C), and low IL-6 expression only correlated with PFS (*P* = 0.02) in the validation cohort (Supplementary Fig. S6B,D).

When we performed combined analysis of Treg cell frequencies and TGF-β mRNA expression, the differences in PFS and OS were more prominent. In the discovery cohort, patients with both a high level of Treg cells and high expression of TGF-β had significantly longer PFS (*P* = 0.007) and OS (*P* = 0.01) compared with both the low groups (Fig. 3G, H). Of note, both high levels of Treg cells and high expression of TGF-β were associated with a high rate of response to anti-PD-1 therapy compared with both low groups 57.1% (12/21) vs 10.5% (2/19) (Fig. 3I). Consistent results were observed in the validation cohort, where patients with high levels of Treg cells and TGF-β had prolonged PFS (*P* = 0.005) and OS (*P* = 0.004) (Fig. 3J, K). Again, the groups of patients with high Treg cells and TGF-β achieved a higher response rate than both low groups 90.9% (10/11) vs 30% (3/10) (Fig. 3L).

### Plasma cytokine levels correlate with Treg cells and clinical outcomes

**Figure 4:**
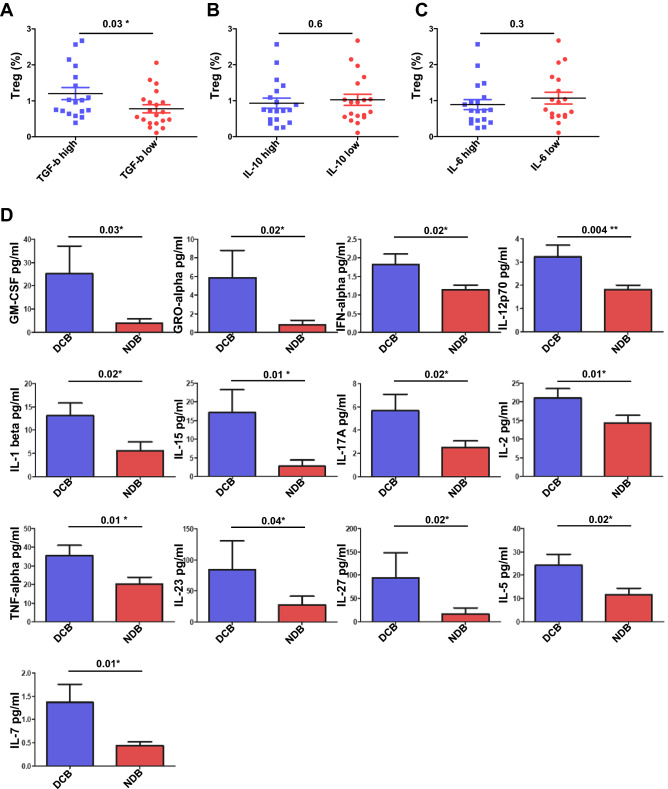
Cytokine protein levels and correlation with Treg cell frequencies in the validation cohort (*n* = 49). Treg cell frequencies in association with (**A**) TGF-β, (**B**) IL-10, or (**C**) IL-6 at a low or high level. (**D**) GM-CSF, GRO-alpha, IFN-alpha, IL-12p70, IL-1beta, IL-15, IL-17A, IL-2, TNF-alpha, I-23, IL-27, IL-5, and IL-7 cytokines in plasma associated with durable clinical benefiters (DCB) or non-durable clinical benefiters (NDB). The center value is mean ± SEM, and the error bar represents the standard deviation of the mean.

Next, we analyzed TGF-β, IL-10, and IL-6 cytokine levels from plasma obtained one week after anti-PD-1 immunotherapy. The median value of TGF-β was 7.5 pg/ml (range 0–38.54 pg/ml). We separated the group into high and low TGF-β levels and analyzed their correlation with Treg cells (%). Only high levels of TGF-β correlated with Treg cells (*P* = 0.03) (Fig. 4A–C). In addition to these three cytokines, we analyzed 34 cytokines and chemokines released during immune response. Among the 34 cytokines and chemokines, high levels of GM-CSF (*P* = 0.03), GRO-alpha (*P* = 0.02), IFN-α (*P* = 0.02), IL-12p70 (*P* = 0.004), IL-1 β (*P* = 0.02), IL-15 (*P* = 0.01), IL-17R (*P* = 0.02), IL-2 (*P* = 0.01), TNF-α (*P* = 0.01), IL-23 (*P* = 0.04), IL-27 (*P* = 0.02), IL-5 (*P* = 0.02), and IL-7 (*P* = 0.01) were associated with an increase in durable clinical benefit from anti-PD-1 immunotherapy (Fig. 4D).

## Discussion

The accumulation of Treg cells in the tumor bed of various cancers is associated with poor prognosis, as expected by their function of inhibiting antitumor immunity and maintaining immune homeostasis. However, high Treg cell frequency in several cancer types can have the opposite result; high Treg cell infiltration is associated with a favorable prognosis in patients with colorectal cancer or ER^-^ breast cancer^13,15^. In a previous study, we found that the frequency of Treg cells increases in NSCLC patients with disease progression just like other suppressive immune cells^18^. However, unlike other suppressive immune cells, high frequencies of Treg cells were consistently associated with prolonged survival. Therefore, we focused on Treg cells in this study and analyzed their correlation with clinical outcomes before and after anti-PD-1 immunotherapy. In this study, we found that high frequencies of circulating Treg cells one week after anti-PD-1 immunotherapy in patients with advanced NSCLC were associated with prolonged PFS, OS, and durable clinical benefit when compared with patients with a low frequency of Treg cells, which is consistent with previous results. Accordingly, high expression of TGF-β is correlated with high levels of Treg cells and is associated with favorable clinical outcomes. Our findings indicate that a high frequency of Treg cells along with a high level of TGF-β one week after anti-PD-1 immunotherapy could be used as favorable blood-based biomarkers at an early point in the therapy of advanced NSCLC.

Recent reports have demonstrated that a higher frequency of circulating or tumor site Treg cells is associated with a favorable response. Similar to our results, Lukesova et al. showed that high frequencies of Treg cells could be used as a positive prognostic marker in patients with HPV-positive oral and oropharyngeal squamous cell carcinomas^14^, and Correale et al. analyzed high Treg cells in patients with advanced colon cancer undergoing chemo and chemoimmunotherapy as a favorable prognostic factor^13^. In addition, tumor-infiltrating Tregs were associated with cytotoxic immune responses and prolonged survival in estrogen receptor-negative breast cancer^15^. In a recent study, Kim et al. found that a high Treg cell/PMN-MDSC ratio in blood correlated with clinical outcomes in NSCLC after anti-PD-1 immunotherapy^19^. Collectively, Treg cells might have different roles in different tumor types, for example, in HPV-positive or -negative oral squamous cell carcinoma and in ER-positive or -negative breast cancer.

One explanation for the favorable outcomes associated with high levels of Treg cells could be the specific task that Treg cells have in preventing autoimmunity and attenuating a potentially dangerous over-reactive immune response after prolonged immune reactions. Treg cell expansion is strictly dependent upon IL-2 levels produced by specific antigen-activated effector lymphocytes either CD4 or CD8^+^ T cells^20,21^. Based on these results, we hypothesize that Treg cell infiltration in tumor tissues as well as high frequencies of circulating Treg cells might be an indirect and powerful indicator of an antitumor immune response to prevent a prolonged non-specific immune reaction once the immune system is activated.

In this study, we also observed that increased mRNA and plasma levels of TGF-β correlated with Treg cell frequencies, and the patient group with high TGF-β correlated with prolonged PFS and OS compared with the low group one week after anti-PD-1 therapy. As a pleiotropic cytokine, TGF-β plays critical roles in Treg cell proliferation and differentiation and regulates adaptive immunity components, such as T cells, as well as the innate immune system^16,21^. Therefore, high levels of TGF-β released by Treg cells may induce the proliferation and activation of Treg cells.

In plasma one week after anti-PD-1 immunotherapy, we observed increased levels of various cytokines involved in the active immune response in patients who achieved durable clinical benefits. These results are consistent with previous studies, which indicate that cytokines are directly or indirectly involved in Treg cell development and maturation, such as GM-CSF, IL12p70, IL-2, and IL-15, are increased in patients who have benefited from anti-PD-1 therapy^22,23^. In addition, cytokines involved in the inflammatory response, including IL-17A, TNF-α, and IL-23, were at high levels in patients with durable clinical benefit^24^. These results suggest that increased levels of cytokines after immune response may attract Treg cells to function as a key factor in immune homeostasis.

Although Treg cells and TGF-β consistently showed favorable outcomes in this study, there are limitations. TGF-β is released from Treg cells, however, other cells such as stromal cells and MDSCs also release TGF-β^25^. Therefore, further analysis of TGF-β expression directly released from Treg cells is needed. In this study, we analyzed FoxP3 from cryopreserved PBMCs, not from fresh PBMCs. However, there was no difference in the FoxP3 expression in CD4^+^CD25^+^CD45RA^-^ cells between fresh and cryopreserved PBMCs (*n* = 7, Supplementary Fig. S7). Given that the frequencies of Treg cells and the expression of TGF-β were associated with a more prominent impact on clinical outcome, Treg cell or TGF-β alone might not be sufficient as a single biomarker to predict the response of therapy in other solid tumors.

In conclusion, our results suggest high levels of circulating CD4^+^CD25^+^CD45RA^-^FoxP3^+^ T cells (effector Treg cells) one week after immunotherapy predict a favorable response in NSCLC patients who were treated with either pembrolizumab or nivolumab anti-PD-1 immunotherapy. Together with Treg cells, the detection of a high TGF-β level also identifies a more favorable outcome, which may specifically benefit from anti-PD-1 immunotherapy. An understanding of the clinical relevance of the tumor microenvironmental immunologic milieu might provide an important clue when designing novel strategies in cancer immunotherapy, however, blood-based Treg cells might also provide alternative sources when a tumor biopsy is not available. Therefore, Treg cell frequency and TGF-β could be used as blood-based biomarkers to predict the anti-PD-1 immunotherapy response in patients with advanced NSCLC.

## Material and methods

### Patients and blood sample collection

Patients with NSCLC (stage IIIB to IV) undergoing anti-PD-1 immunotherapy with either pembrolizumab (200 mg every 3 weeks) or nivolumab (2 mg/kg every 2 weeks) were enrolled in part of a phase II clinical trial (NCT02607631) at the Samsung Medical Center (South Korea). At baseline and one week after anti-PD-1 therapy, peripheral blood samples were collected from March 2017 to February 2018 for the discovery cohort (*n* = 83) and March 2018 to March 2019 for the validation cohort (*n* = 49). All discovery and validation cohort patients had no previous history of immune checkpoint inhibitor treatment. Baseline blood was collected before the first-line therapy and the post-therapy blood sample was collected one week after the therapy. This study was conducted in accordance with the principles of the Declaration of Helsinki and Good Clinical Practice. All protocols were approved by the Institutional Review Board of Samsung Medical Center and all patients signed informed consent. All studies involving patients were conducted in accordance with the ethical guidelines stated by the Samsung Medical Center. The characteristics of the patients are shown in Table 1. Patients who benefitted from anti-PD-1 longer than 6 months (continued treatment without disease progression) were defined as durable clinical benefiters (DCB) and for less than 6 months as non-durable benefiters (NDB). Patients with DCB were defined as responders, and patients with NDB were defined as non-responders.

### Blood preparation

PBMCs were isolated from the whole blood by density centrifugation using Ficoll Paque (GE Healthcare, Chicago, IL, USA) mixed with 1:1 of PBS, at 400×*g* for 25 min at room temperature. Isolated PBMCs were washed with RPMI (Gibco, Thermo Fisher Scientific, Waltham, MA, USA) at 400×*g* for 10 min at 4 °C. PMN-MDSCs and M-MDSCs were analyzed on the same day of PBMC isolation and for Treg cells, PBMCs were cryopreserved for later use. For plasma sample preparation, 10 ml of whole blood was collected from the patients. Blood samples were then centrifuged at 1500×*g* for 10 min at 4 °C and the plasma layer was collected and stored at -70 °C until use.

### Flow cytometry analysis

For Treg cells, isolated PBMCs were stained with anti-CD4-FITC (RPA-T4/555346), CD25-APC (M-A251/555434), and CD45RA-PerCP-Cy 5.5 (HI100/563429) antibodies (BD Biosciences, San Jose, CA, USA) for 45 min, and antibody stained samples were washed twice. After intracellular staining, Treg cell frequencies were analyzed by a BD FACSVerse (BD Biosciences) flow cytometer. For MDSCs, isolated PBMCs were stained with anti-CD3-BV421 (UCHT1/562426), CD19-BV421 (HIB19/562440), CD56-BV421 (NCAM16.2/562751), CD20-BV421 (2H7/562873), CD11b-BB515 (ICRF44/564517), CD15-PerCP-Cy 5.5 (HI98/560828), CD14-APC (M5E2/555399), and HLA-DR-PE (G46-6/555812) antibodies (BD Biosciences) for 45 min, washed twice, and analyzed by a BD FACSVerse (BD Biosciences) flow cytometer. For 7-AAD and propidium iodide staining, isolated PBMCs were stained with 7-AAD (Biolegend, San Diego, CA, USA) or PI (BD Biosciences) for 10 min and then analyzed on a BD FACSVerse (BD Biosciences). Gating strategies are shown in Supplementary Fig. S1. PBMC viability before MDSC analysis is shown in Supplementary Fig. S2.

### Intracellular staining

After PBMCs were stained with cell surface markers, cells were fixed and permeabilized with TF fix/perm for 40 min and then washed with Perm Wash Buffer (BD Biosciences). Cells were then stained with Foxp3-PE (259D/C7/560046) (BD Biosciences) for 45 min. Samples were washed twice with Perm Wash Buffer and then analyzed by BD FACSVerse (BD Biosciences).

### mRNA expression—real-time quantitative PCR

To measure TGF-β, IL-10, and IL-6 mRNA expression, we isolated total RNA from PBMCs using an RNeasy Mini Kit (Qiagen, Hilden, Germany). cDNA was then constructed from total RNA using the Superscript III first-strand synthesis system (Invitrogen, Carlsbad, CA, USA) according to the manufacturer’s instructions. TGF-β 1, IL-10, IL-6, and β-actin TaqMan Gene Expression Assays and TaqMan Gene Expression Master Mix (Thermo Fisher Scientific, Waltham, MA, USA) were used for RT PCR, and gene expression was measured with an Applied Biosystem PRISM 7900HT (384-well mode) (Applied Biosystem, Foster City, CA, USA) and analyzed by SDS2.4 software^18^.

### Protein expression—ELISA

To measure TGF-β, IL-10, and IL-6 protein expression, a human TGF-β1 Quantikine ELISA Kit, Human IL-10 Quantikine ELISA Kit, and Human IL-6 Quantikine ELISA Kit (R&D Systems, Minneapolis, MN, USA) were used according to the manufacturer’s instructions. Cytokine levels were measured using a SPECTRA max plus microplate reader set to 450 nm (Molecular Devices, San Jose, CA, USA) and analyzed by GraphPad Prism 5 (GraphPad, La Jolla, CA, USA).

### Cytokine and chemokine protein assay and analysis

To measure 34 cytokines and chemokine in the plasma of the patients, Cytokine & Chemokine 34-Plex Human ProcartaPlex Panel 1A was used (Invitrogen, Carlsbad, CA, USA) according to the manufacturer’s instructions. Cytokine levels were measured using a BioPlex 200 System (Bio-Rad Laboratories, Hercules, CA, USA) and analyzed by GraphPad Prism 5 (GraphPad, La Jolla, CA, USA).

### Statistical analyses

Data were analyzed by independent two-tailed Student’s t-tests or Mann–Whitney *U* test with 95% confidence intervals. If the data did not follow normal distribution by the Shapiro–Wilk normality test, the Mann–Whitney *U* test was used for two independent groups. Survival curves were obtained using the Kaplan–Meier method and comparisons were made using the log-rank (Mantel-Cox) test. All statistical analyses were performed with GraphPad Prism 8 (GraphPad) two-tailed *P* values < 0.05 were considered significant.

## Supplementary information

Supplementary Information

## Data Availability

The datasets generated and analyzed during the current study are available from the corresponding author on reasonable request.

## Abbreviations

Treg cell: Regulatory T cell
TGF-β: Transforming growth factor-beta
PMN-MDSC: Polymorphonuclear-myeloid-derived suppressor cell
M-MDSC: Monocytic-myeloid-derived suppressor cell
IL: Interleukin

## References

[1] Villaruz LC, Kalyan A, Zarour H, Socinski MA (2014). Immunotherapy in lung cancer. Transl. Lung Cancer Res..

[2] Wu X (2019). Application of PD-1 blockade in cancer immunotherapy. Comput. Struct. Biotechnol. J..

[3] Berghmans T, Durieux V, Hendriks LEL, Dingemans AM (2020). Immunotherapy: From advanced NSCLC to early stages, an evolving concept. Front. Med. Lausanne.

[4] Sui H (2018). Anti-PD-1/PD-L1 therapy for non-small-cell lung cancer: Toward personalized medicine and combination strategies. J. Immunol. Res..

[5] Cho JH (2017). Immunotherapy for non-small-cell lung cancer: current status and future obstacles. Immune Netw..

[6] Meyer C (2014). Frequencies of circulating MDSC correlate with clinical outcome of melanoma patients treated with Ipilimumab. Cancer Immunol. Immunother..

[7] Beury DW (2014). Cross-talk among myeloid-derived suppressor cells, macrophages, and tumor cells impacts the inflammatory milieu of solid tumors. J. Leukoc. Biol..

[8] Wei SC, Duffy CR, Allison JP (2018). Fundamental mechanisms of immune checkpoint blockade therapy. Cancer Discov..

[9] Sakaguchi S (2005). Naturally arising Foxp3-expressing CD25^+^CD4^+^ regulatory T cells in immunological tolerance to self and non-self. Nat. Immunol..

[10] Sakaguchi S, Sakaguchi N, Asano M, Itoh M, Toda M (1995). Immunologic self-tolerance maintained by activated T cells expressing IL-2 receptor alpha-chains (CD25). Breakdown of a single mechanism of self-tolerance causes various autoimmune diseases. J. Immunol..

[11] Liang J (2019). FOXA1(+) regulatory T cells: A novel T cell subset that suppresses antitumor immunity in lung cancer. Biochem. Biophys. Res. Commun..

[12] Kotsakis A (2016). Prognostic value of circulating regulatory T cell subsets in untreated non-small cell lung cancer patients. Sci. Rep..

[13] Correale P (2010). Regulatory (FoxP3+) T-cell tumor infiltration is a favorable prognostic factor in advanced colon cancer patients undergoing chemo or chemoimmunotherapy. J. Immunother..

[14] Lukesova E (2014). High level of Tregs is a positive prognostic marker in patients with HPV-positive oral and oropharyngeal squamous cell carcinomas. Biomed. Res. Int..

[15] West NR (2013). Tumour-infiltrating FOXP3(+) lymphocytes are associated with cytotoxic immune responses and good clinical outcome in oestrogen receptor-negative breast cancer. Br. J. Cancer.

[16] Wan YY, Flavell RA (2007). 'Yin-Yang' functions of transforming growth factor-beta and T regulatory cells in immune regulation. Immunol. Rev..

[17] Oh SA, Li MO (2013). TGF-β: Guardian of T cell function. J. Immunol..

[18] Koh, J. *et al.* MDSC subtypes and CD39 expression on CD8(+) T cells predict the efficacy of anti PD-1 immunotherapy in patients with advanced NSCLC. *Eur. J. Immunol.* (2020). 10.1002/eji.202048534 PMC7689686 32510574

[19] Kim HR (2019). The ratio of peripheral regulatory T cells to Lox-1(+) polymorphonuclear myeloid-derived suppressor cells predicts the early response to anti-PD-1 therapy in patients with non-small cell lung cancer. Am. J. Respir. Crit. Care Med..

[20] Sojka DK, Huang YH, Fowell DJ (2008). Mechanisms of regulatory T-cell suppression—A diverse arsenal for a moving target. Immunology.

[21] Romano M, Fanelli G, Albany CJ, Giganti G, Lombardi G (2019). Past, present, and future of regulatory T cell therapy in transplantation and autoimmunity. Front. Immunol..

[22] Kwon KW (2019). IL-15 Generates IFN-γ-producing cells reciprocally expressing lymphoid-myeloid markers during dendritic cell differentiation. Int. J. Biol. Sci..

[23] Zelante T, Fric J, Wong AY, Ricciardi-Castagnoli P (2012). Interleukin-2 production by dendritic cells and its immuno-regulatory functions. Front Immunol..

[24] Kuwabara T, Ishikawa F, Kondo M, Kakiuchi T (2017). The Role of IL-17 and related cytokines in inflammatory autoimmune diseases. Mediat. Inflamm..

[25] Dahmani A, Delisle JS (2018). TGF-β in T Cell Biology: Implications for cancer immunotherapy. Cancers (Basel).

