# Supplementary material for: Regulatory (FoxP3+) T cells and TGF-β predict the response to anti-PD-1 immunotherapy in patients with non-small cell lung cancer
Source: Sci Rep. 2020 Nov 4;10:18994. doi: 10.1038/s41598-020-76130-1 (PMC7642363; doi:10.1038/s41598-020-76130-1)
Supplement: Supplementary file 1 — Supplementary Information [file 41598_2020_76130_MOESM1_ESM.docx]

**Supplementary Data**

**Regulatory (FoxP3^+^) T cells and TGF-β predict the response to anti-PD-1 immunotherapy in patients with non-small cell lung cancer**

Jiae Koh^1, 3*^, Joon Young Hur^2,4*^, Kyoung Young Lee^3^, Mi Soon Kim^3^, Jae Yeong Heo^3^, Bo Mi Ku^3^, Jong-Mu Sun^2^, Se-Hoon Lee^1,2^, Jin Seok Ahn^2^, Keunchil Park^2^, Myung-Ju Ahn^1,2^

^1^Department of Health Sciences and Technology, SAIHST, Sungkyunkwan University, Seoul, Korea; ^2^Division of Hematology-Oncology, Department of Medicine, Samsung Medical Center, Sungkyunkwan University School of Medicine, Seoul, Korea; ^3^Research Institute for Future Medicine, Samsung Medical Center, Sungkyunkwan University School of Medicine, Seoul, Korea; ^4^Division of Hematology and Oncology, Department of Internal Medicine, Hanyang University Guri Hospital, Guri, South Korea

*These authors contributed equally to this work as first authors.

Correspondence to:

***Prof. Myung-Ju Ahn**, Division of Hematology-Oncology, Department of Medicine, Samsung Medical Center, Sungkyunkwan University School of Medicine; Department of Health Sciences and Technology, SAIHST, Sungkyunkwan University, 81 Irwon-ro, Gangnam-gu, Seoul, 06351, Republic of Korea; Tel: +82-2-3410-3438; Fax: +82-2-3410-1754; Email: [silkahn@skku.edu](mailto:silkahn@skku.edu)

**Supplementary Figures**

**Supplementary Figure S1.**


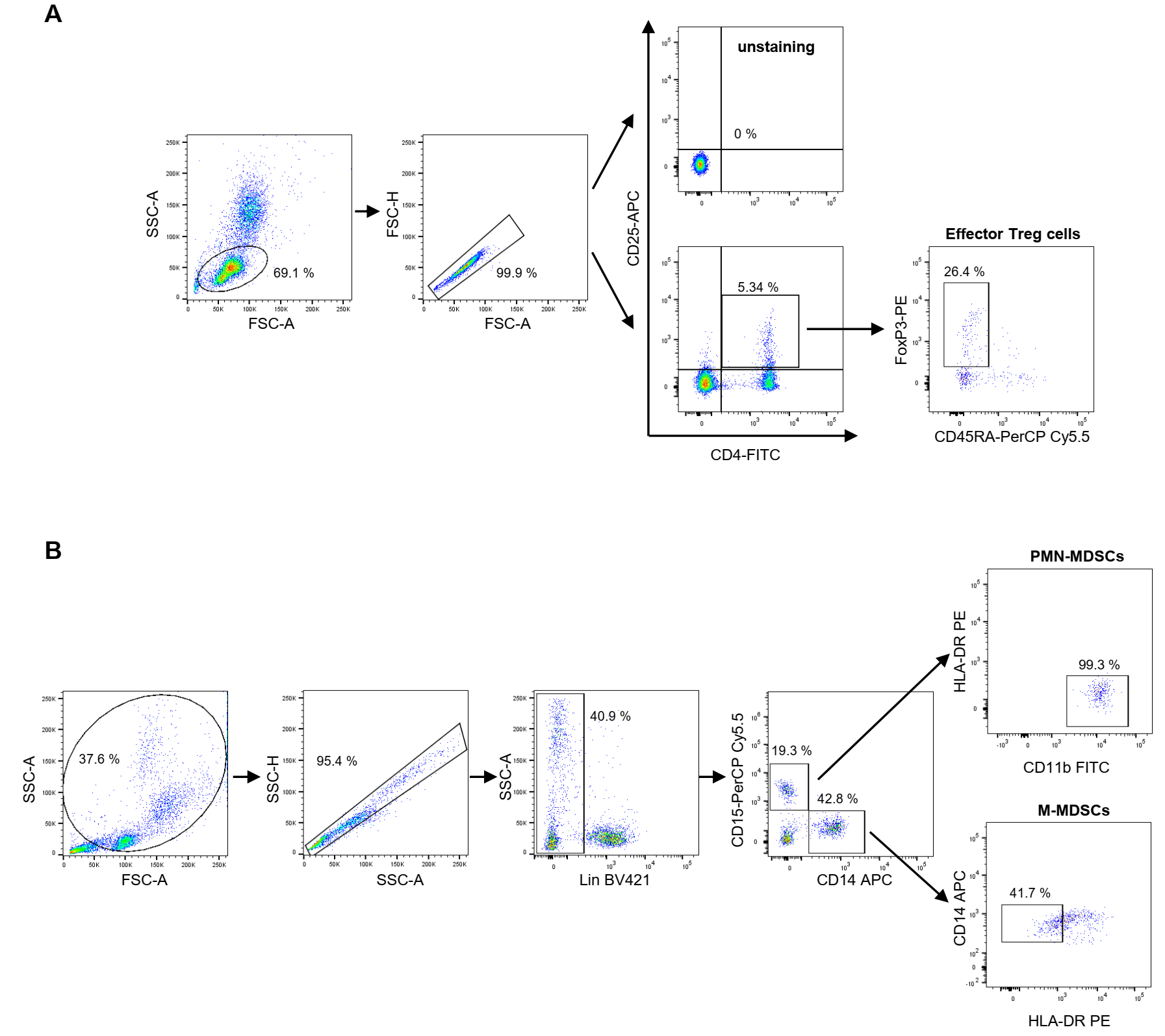


**Supplementary Figure S1.** Gating strategies for effector Treg cells and myeloid-derived suppressor cells (MDSCs) measured by flow cytometer. (**A**) The representative gating strategies for effector Treg cells (CD4^+^CD25^+^CD45RA^-^FoxP3^+^). Lymphocytes were gated from whole peripheral blood mononuclear cells (PBMCs) and then single cells were gated. CD4^+^CD25^+^ cells were gated and then CD45RA^-^FoxP3^+^ cells were gated. (**B**) The representative gating strategies for polymorphonuclear (PMN)-MDSCs and monocytic (M)-MDSCs. All leukocytes and then single cells were gated. Lineage (-) which are CD3, CD4, CD8, CD19, CD20, and CD56 all negative cells were gated. For PMN-MDSCs, CD15^+^CD14^-^ cells were gated, and then CD11b^+^ and HLA-DR^-/low^ cells were gated. For M-MDSCs, CD14^+^ CD15^-^ and HLA-DR^-/low^ cells were gated from lineage negative cells.

**Supplementary Figure S2.**


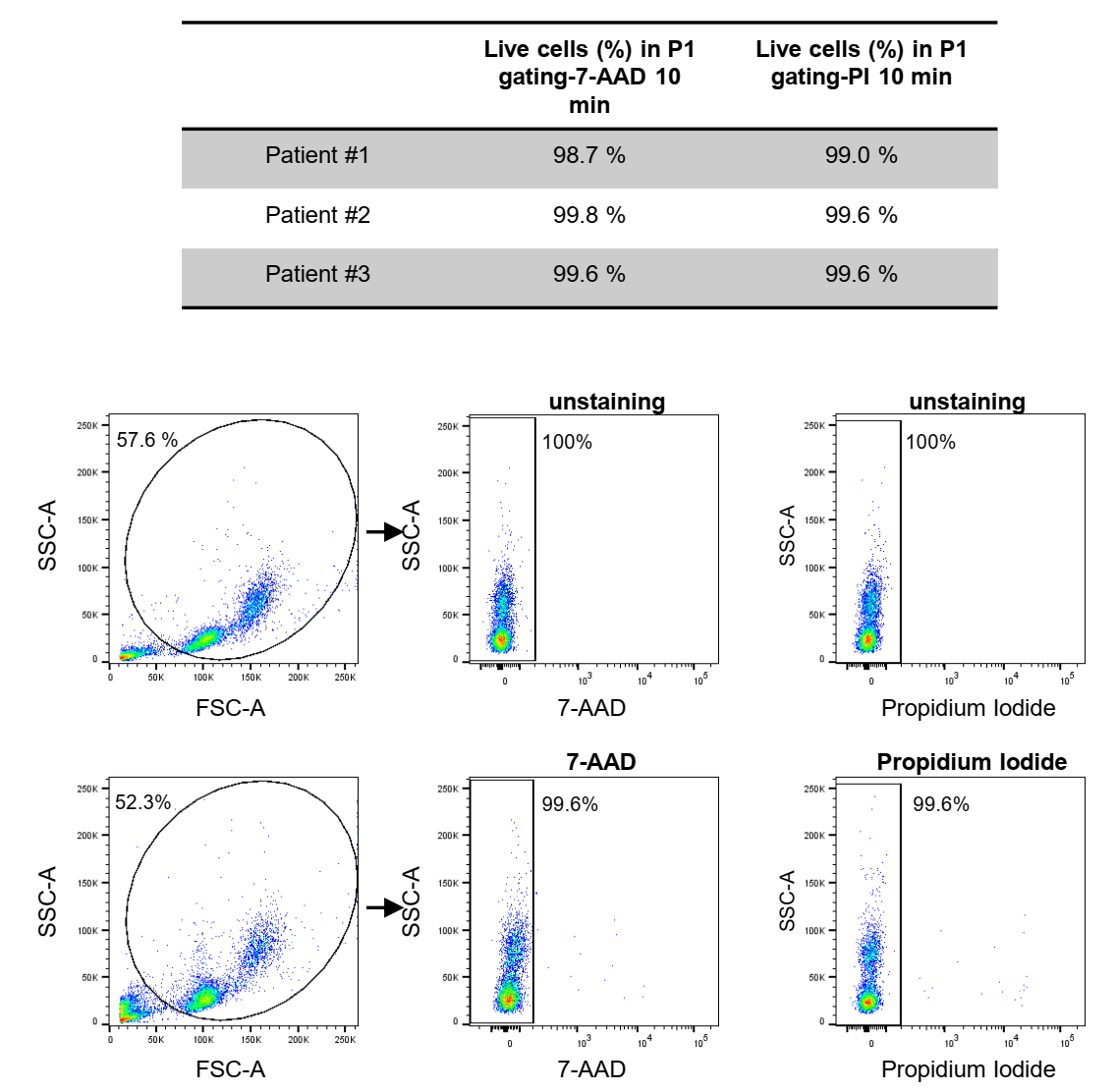


**Supplementary Figure S2.** Isolated peripheral blood mononuclear cells (PBMCs) viability before myeloid-derived suppressor cells (MDSCs) analysis. For live cells (%), all leukocytes from PBMCs were gated and then 7-AAD or propidium iodide negative cells were gated (*n* = 3).

**Supplementary Figure S3.**


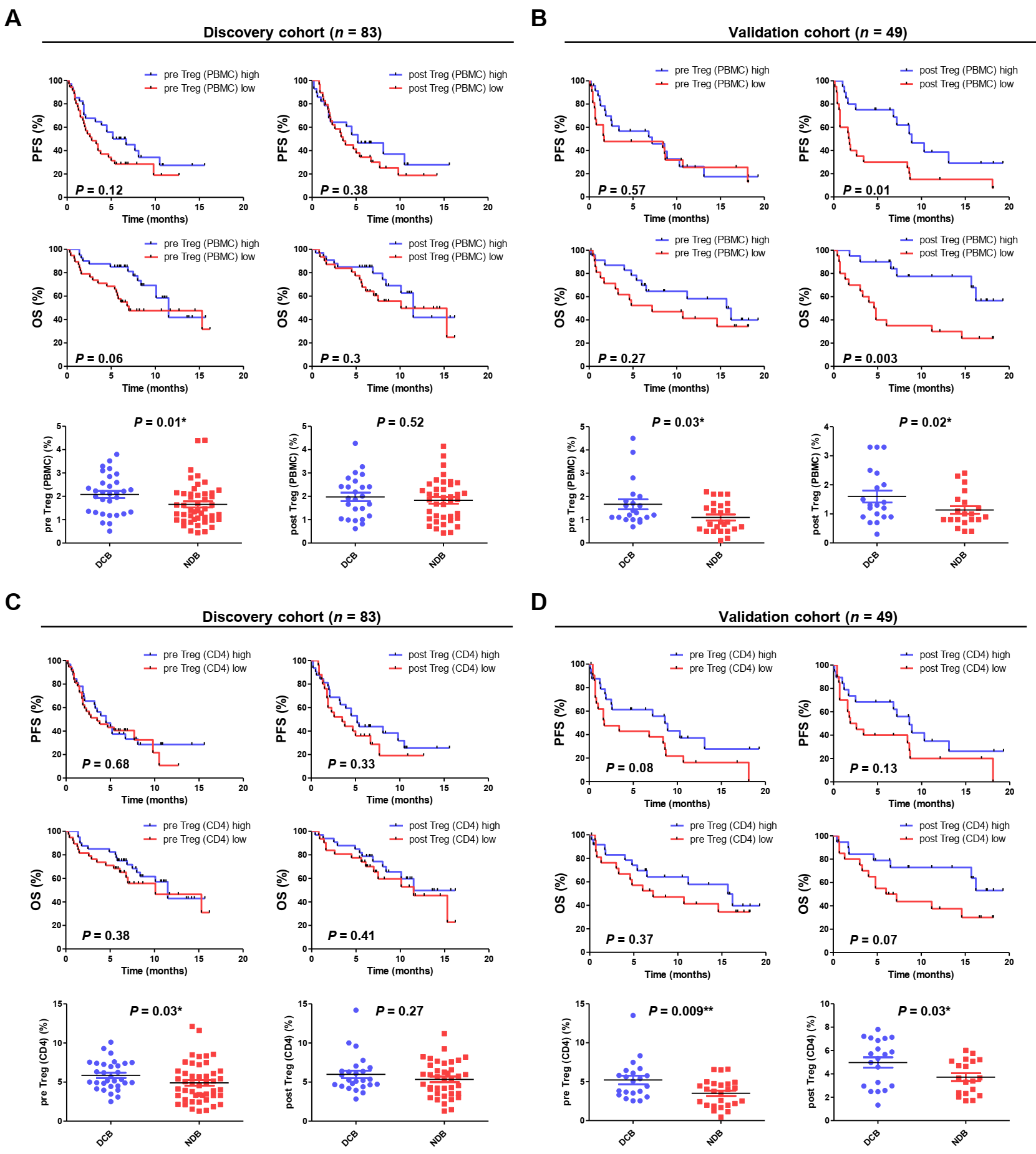


**Supplementary Figure S3.** Clinical outcomes depending on Treg cell frequencies. **(A)** Progression-free survival (PFS) and overall survival (OS) of the patients with Treg cells (% of CD4^+^CD25^+^FoxP3^+^ in peripheral blood mononuclear cells, PBMCs) high or low before and after anti-PD-1 therapy, and Treg cells (%) depending on the patients with DCB or NDB before and after anti-PD-1 therapy in the discovery cohort (*n* = 83). (**B**) PFS and OS of the patients with Treg cells (% of CD4^+^CD25^+^FoxP3^+^ in PBMC) high or low before and after anti-PD-1 therapy, and Treg cells (%) depending on the patients with DCB or NDB before and after anti-PD-1 therapy in the validation cohort (*n* = 49). **(C)** PFS and OS of the patients with high or low frequencies of Treg cells (% of CD4^+^CD25^+^FoxP3^+^ in CD4^+^ T cells) before and after anti-PD-1 therapy. Treg cells (%) depending on the patients with DCB or NDB before and after anti-PD-1 therapy in the discovery cohort. (**D**) PFS and OS of the patients with high or low frequencies of Treg cells (% of CD4^+^CD25^+^FoxP3^+^ in CD4^+^ T cells) before and after anti-PD-1 therapy. Treg cells (%) depending on the patients with DCB or NDB before and after anti-PD-1 therapy in the validation cohort. For CD4^+^CD25^+^FoxP3^+^ Treg cells (% in PBMC) before the therapy, the median cutoff values of pre-Treg cells are 1.75% (range: 0.44-4.4) in discovery cohort and 1.2% (range: 0.13–4.47) in validation cohort. After the therapy, the median cutoff values of post-Treg cells are 1.9% (range: 0.43–4.27) in the discovery cohort and 1.13% (range: 0.34-3.33) in the validation cohort. For CD4^+^CD25^+^FoxP3^+^ Treg cells (% in CD4^+^ T cells) before the therapy, the median cutoff values of pre-Treg cells are 5.03% (range: 1.28–12.1) in discovery cohort and 3.99% (range: 0.46–13.5) in the validation cohort. After the therapy, the median cutoff values of post-Treg cells are 5.27% (range: 1.28–14.2) in the discovery cohort and 4.63% (range: 1.33–7.81) in the validation cohort. The center value is mean±SEM. Kaplan-Meier survival curves of patients were plotted with a median cutoff. Statistical significance was determined by log-rank (Mantel-Cox) regression analysis, with the level of significance at *P* $\leq$ 0.05.

**Supplementary Figure S4.**


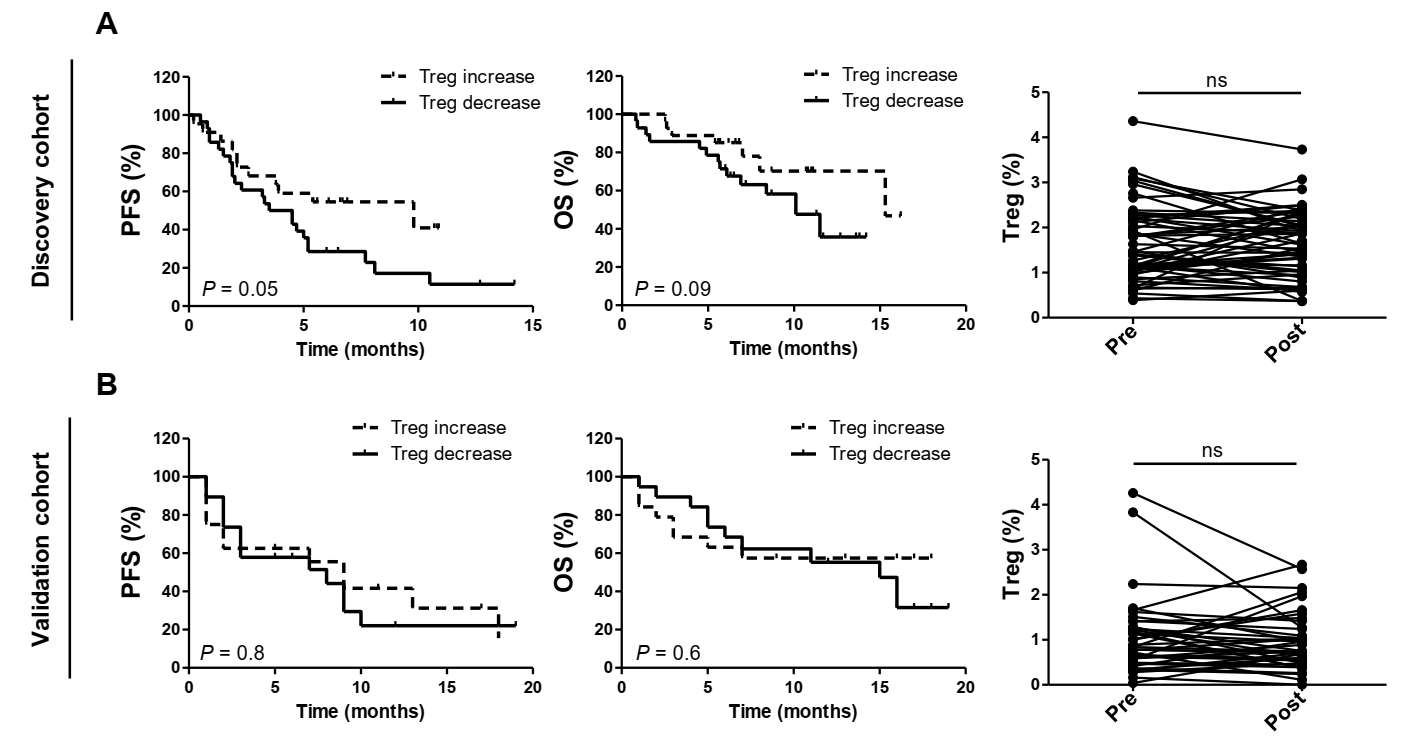


**Supplementary Figure S4.** Progression-free survival (PFS) and overall survival (OS) of patients with advanced NSCLC in patient groups with increased or decreased Treg cells. (**A**) PFS and OS depending on patient groups with increased or decreased Treg cells after anti-PD-1 therapy. Treg cells (%) of each patient before and after the therapy in the discovery cohort (*n* = 83). **(B)** PFS and OS depending on whether Treg cells are increased or decreased after anti-PD-1 therapy. Treg cells (%) of each patient before and after the therapy in the validation cohort (*n* = 49). Kaplan-Meier survival curves were plotted with a median cutoff. Statistical significance was determined by log-rank (Mantel-Cox) regression analysis, with the level of significance at *P* $\leq$ 0.05.

**Supplementary Figure S5.**


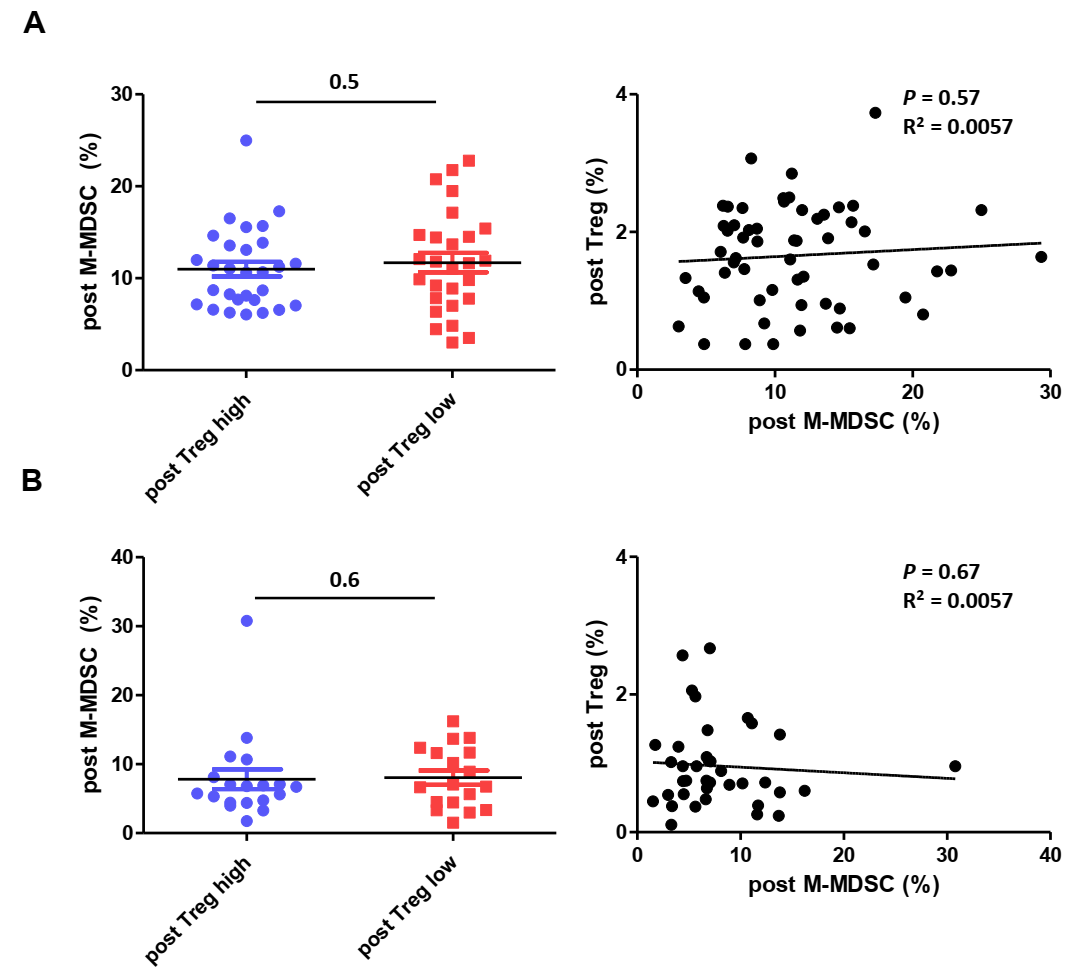


**Supplementary Figure S5.** Correlation of Treg cells and monocytic-myeloid-derived suppressor cells (M-MDSCs). (**A**) M-MDSCs frequencies that are associated with high or low levels of Treg cells and coefficient correlation of Treg cells and M-MDSC frequencies in the discovery cohort. (**B**) M-MDSC frequencies that are associated with high or low levels of Treg cells and the coefficient correlation of Treg cells and M-MDSC frequencies in the validation cohort. The center value is the mean±SEM. Kaplan-Meier survival curves were plotted with a median cutoff. Statistical significance was determined by log-rank (Mantel-Cox) regression analysis, with the level of significance at *P* $\leq$ 0.05.

**Supplementary Figure S6.**


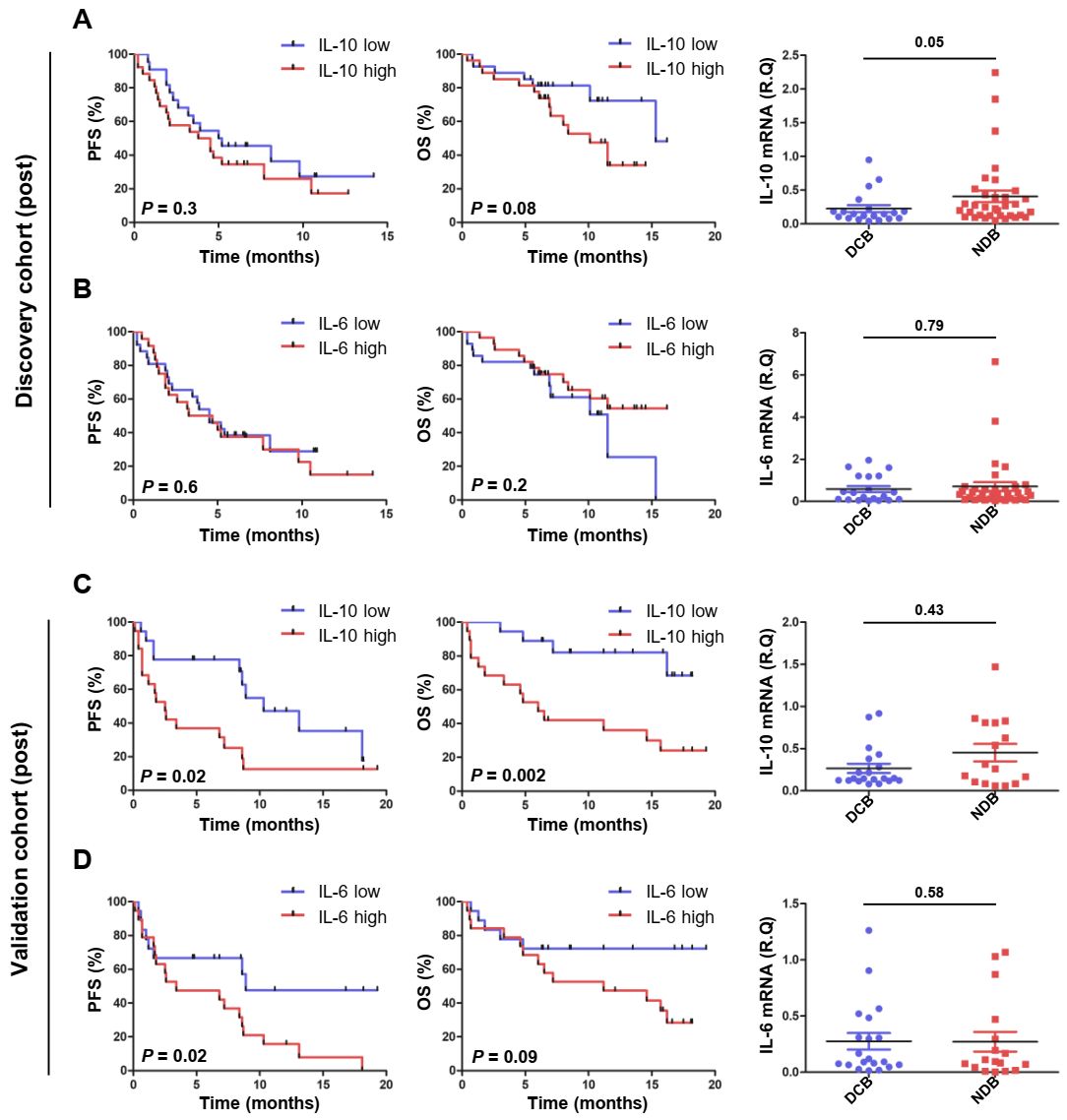



**Supplementary Figure S6.** Cytokine mRNA level correlation with clinical outcomes. (**A**) Progression-free survival (PFS), overall survival (OS), and durable clinical benefiters depending on low or high levels of IL-10 in the discovery cohort (*n* =83). (**B**) PFS, OS, and durable clinical benefiters depending on low or high levels of IL-10 in the validation cohort (*n* = 49). (**C**) PFS, OS, and durable clinical benefiters depending on low or high levels of IL-6 in the discovery cohort (*n* =83). (**D**) PFS, OS, and durable clinical benefiters depending on low or high levels of IL-6 in the validation cohort (*n* = 49). The center value is the mean±SEM. Kaplan-Meier survival curves were plotted with patients by median cutoff. Statistical significance was determined by log-rank (Mantel-Cox) regression analysis, with the level of significance at *P* $\leq$ 0.05.

**Supplementary Figure S7.**

**
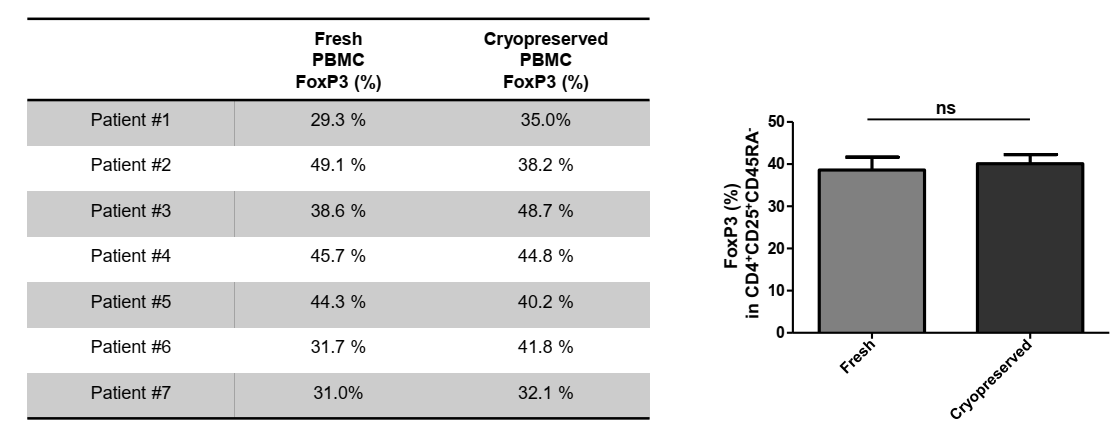
**

**Supplementary Figure S7.** Comparison of FoxP3 expression in CD4^+^CD25^+^CD45RA^-^ T cells from fresh peripheral blood mononuclear cells (PBMCs) and cryopreserved PBMCs (*n* = 7). The error bar represents the standard deviation of the mean.
